# Effects of hot water extract of Juncao-substrate *Ganoderma lucidum* residue on meat quality and antioxidant capacity of Liancheng white ducks

**DOI:** 10.1016/j.psj.2026.106822

**Published:** 2026-03-18

**Authors:** Zai-Xing Cai, Hai-Xuan Lv, Yun Yang, Xiao-Ming Gu, Xiao-Ping Liu, Ling Jin, Yu-Yun Gao

**Affiliations:** a College of Animal Sciences, Fujian Agriculture and Forestry University, Fuzhou 350002, China; b China National Engineering Research Center of JUNCAO Technology, Fujian Agriculture and Forestry University, Fuzhou 350002, China

**Keywords:** *Ganoderma lucidum* polysaccharides, Liancheng white duck, Production Performance, Antioxidant, Lipid metabolism

## Abstract

The present study explored the effects of dietary supplementation with hot water extract of Juncao-substrate *Ganoderma lucidum* residue (HWE-JGLR) on growth performance, carcass traits, meat quality, and antioxidant capacity of Liancheng white ducks. A total of 288 one-day-old male Liancheng white ducks were randomly allocated into 4 groups with 6 replicates of 12 ducks each. The control group was fed a corn-soybean meal basal diet, while the test groups were fed a basal diet supplemented with either 0.25%, 0.5%, or 1% HWE-JGLR, designated as HJ-I, HJ-Ⅱ, and HJ-Ⅲ groups, respectively, for 63 days. No significant differences were observed in growth performance or carcass traits among the groups. Regarding meat quality, the primary beneficial effect was the mitigation of pH decline in breast muscles (*P* < 0.05); no significant effects were observed in shear force, drip loss, or meat color of both breast and leg muscles. In breast muscle, the expression levels of genes *CPT-1* and PRKAA1 were elevated by HWE-JGLR supplementation (*P* < 0.05). In contrast, a marked suppression was observed in the expression of *SREBP-1C* (*P* < 0.05). Additionally, compared with the control group, the serum T-SOD and GSH-Px activities in ducks fed HJ-Ⅱ and HJ-Ⅲ diets were found to be increased (*P* < 0.05). Besides, ducks fed HJ-I and HJ-Ⅲ diets reduced serum MDA concentration (*P* < 0.05). In liver and breast muscle, HJ-Ⅱ and HJ-Ⅲ groups increased the expression of *CAT, SOD, TXN, GCLC, NRF2* and *NQO1* (*P* < 0.05). Meanwhile, the activity of liver GSH-Px and breast muscle CAT was also enhanced (*P* < 0.05). In summary, diet supplemented with HWE-JGLR has been shown to improve the meat quality of breast muscle and suppress the expression levels of fat-related genes. Meanwhile, HWE-JGLR promoted the antioxidant capacity of Liancheng through a coordinated increase in antioxidant enzyme activities and upregulation of associated gene expression. Based on the results of various measurement indicators, it is appropriate to add 0.5% HWE-JGLR in feed.

## Introduction

Although intensive poultry farming systems effectively meet the demand for poultry products, they also pose significant drawbacks, including adverse effects on the poultry health due to factors such as physiological stress and digestive disorders (Averós and Estevez, 2018). Historically, the incorporation of antibiotics into feed was a conventional strategy to mitigate these challenges (Castanon, 2007). Since 2020, however, China has phased out this practice, aligning with policies in the United States and the European Union by prohibiting the use of antibiotic growth promoters in animal feed (Gao et al., 2023). Consequently, finding alternative strategies to enhance animal production, immunity and disease resistance without relying on antibiotics has emerged as a central challenge for the industry, which is crucial for ensuring its future profitability and sustainability (Li et al., 2022).

*Ganoderma lucidum* is a medicinal mushroom highly valued in traditional Chinese medicine, belonging to the Polyporaceae family of Basidiomycetes (Rousseau, 2021). Chemical analyses have consistently demonstrated that *Ganoderma lucidum* contains elevated concentrations of important bioactive compounds, including polysaccharides, nucleosides, triterpenoids and sterols (Blundell et al., 2023). Juncao refers to herbaceous plants that are specifically cultivated to function as a growth substrate for edible and medicinal fungi (Liu et al., 2022b). Juncao-substrate *Ganoderma lucidum* residue (JGLR) is the spent substrate remaining after the harvest of Ganoderma lucidum fruiting bodies from the Juncao grass-based cultivation medium. Hot water extract of Juncao-substrate *Ganoderma lucidum* residue (HWE-JGLR), a powdered extract obtained through hot water extraction of JGLR, is rich in bioactive components, particularly *Ganoderma lucidum* polysaccharide (GLP), amino acids and other physiologically active compounds (Liu et al., 2015b; Martín et al., 2023). Research has extensively demonstrated that GLP, the primary bioactive component of the HWE-JGLR (Martín, et al., 2023), exhibits a wide range of pharmacological effects, including antibacterial (Cör et al., 2018), antioxidant (Seweryn et al., 2021), antitumor (Kong et al., 2019), hypolipidemic (Wu, 2018), and immunomodulatory activities (Liu et al., 2022a). The multifunctional nature of HWE-JGLR makes it a promising candidate for various applications in animal production, with considerable potential for industry development. Treatment with HWE-JGLR in mice induced a significant immunomodulatory effect, marked by elevated spleen and thymus indices, alongside a potent antioxidative response characterized by increased serum concentrations of SOD, CAT, and T-AOC (Liu et al., 2015c). Moreover, evidence from research confirms that HWE-JGLR supplementation increased milk yield and enhanced the concentrations of the immune component IgG in dairy cows (Liu et al., 2015a, 2015b). Furthermore, our previous research (Gao et al., 2024) has found that HWE-JGLR not only positively modulates the gut microbiota by elevating probiotic abundance in broilers but also consequently enhances their growth performance.

As a rare indigenous breed endemic to China (Li et al., 2023b), the Liancheng white duck is prized not only for egg production but also for the unique flavor, texture and medical value of its meat, contributing to its inclusion in China’s National List of Livestock and Poultry Genetic Resources Protection (Li et al., 2006). Nevertheless, the high cost of *Ganoderma lucidum* or GLP pure product limits its direct use as feed additives in commercial duck production. In contrast, HWE-JGLR represents a more cost-effective alternative that enhances economic returns while minimizing the waste of mushroom substrate resources and promoting environmental sustainability. However, most previous studies have focused on broilers, and little is known about the effects of HWE-JGLR in ducks. Given the physiological differences between ducks and broilers, particularly in lipid metabolism (Hermier, 1997) and meat quality traits (Gornowicz et al., 2023), validation in duck species is essential. Therefore, this study was conducted to investigate the effects of HWE-JGLR on the growth performance and antioxidant capacity of Liancheng white ducks under intensive farming conditions, thereby providing a theoretical basis and technical support for the application of HWE-JGLR in practical production and for improving the health benefits of Liancheng white ducks.

## Materials and methods

### Animal ethics

All the experimental procedures applied in this study were reviewed and approved by the Committee of Animal Experiments of Fujian Agriculture and Forestry University (Fuzhou, Fujian, China, approval ID PZCASFAFU23004).

### Preparation of HWE-JGLR

HWE-JGLR was obtained by hot water extraction from the China National Engineering Research Center of JUNCAO Technology of Fujian Agriculture and Forestry University (Liu et al., 2015b). The high-quality spent Juncao-substrate *Ganoderma lucidum* Residue (JGLR) was selected, dried to constant weight, and then crushed. Through analysis and screening, the powder of JGLR was derived. JGLR was extracted three times at 100 °C: the first and second times were extracted for 2 h with solid-to-liquid ratios of 1:10 and 1:8, respectively, and the third time was extracted for 1.5 h with a ratio of material to solvent of 1:6. The final supernatant was concentrated under the conditions of 0.09 MPa and 55-65 °C. Subsequently, HWE-HGLR was produced through the spray drying process and then stored at 4 °C until use. Proximate analysis revealed that the main components of HWE-JGLR were crude protein (23.58%), crude ash (17.60%), total amino acids (4.95%), and crude fat (0.20%) (Liu et al., 2015b), with a Ganoderma lucidum polysaccharide content of 15.79%, as determined by the procedure described by Nataraj et al. (2023).

### Experimental design and diets

A total of 288 male Liancheng white ducks (Fujian Liancheng White Duck Breeding Farm, Liancheng, Fujian, China) were allocated to 4 experimental groups, using a single-factor completely randomized design, with 6 replicates of 12 ducks each. The basal diet (Jinhua Long Feed Co., Ltd., Fuzhou, China) was prepared in accordance with the Nutrient Requirements of Meat-Type Duck (Chinese Ministry of Agriculture, NY/T 2122-2012), and the diets were formulated through three stages of 1-21 d, 22-49 d and 50-63 d. Ducks in the four treatments were fed a basal diet supplemented with 0, 0.25%, 0.5%, and 1% HWE-JGLR for 63 days. Ingredient composition and nutritional status of the basal diets are presented in Table 1. Before the experiment, the duck facility was meticulously cleaned and disinfected by formaldehyde fumigation to ensure environmental hygiene. Ducks were raised in single-layer cages with 12 birds per cage. Upon arrival at the facility, ducks were maintained at an initial temperature of 32°C. Subsequently, it was reduced by 1°C at 2-3-day intervals until stabilizing at 20°C at 28 days of age. In addition to adequate ventilation and lighting, ducks received twice-daily water spraying to accommodate their aquatic preferences and support natural growth within the cage-rearing system.

**Table 1:** Composition and nutrient levels of basal diets (air-dry basis, %).

Ingredients	Contents (g/kg)		
	1-21 Days	22-49 Days	50-63 Days
Corn	622	670	717
Soybean meal	272	225	167
Extruded soybean	67.7	0	32.8
Wheat bran	0	67.4	47.5
Limestone powder	9.40	10.6	10.2
CaHPO_4_	14.9	12.7	11.8
NaCl	2.50	2.50	2.50
DL-Met	1.30	1.30	1.20
L-lysine hydrochloride (98%)	0.200	0.500	0
Premix^1^	10.0	10.0	10.0
Total	1000	1000	1000
Nutrient levels^2^			
ME (MJ/kg)	12.1	11.7	12.1
CP	200	170	150
Ca	9.00	8.50	8.00
AP	4.20	3.80	3.50
Lys	10.5	8.50	7.10
Met+Cys	7.80	7.00	6.00

Abbreviations: ME, metabolisable energy; CP, crude protein; Ca, calcium; AP, available phosphorus.

1 The premix provided the following per kg of diets: vitamin A, 3 000 IU; vitamin D_3_, 1 200 IU; vitamin E, 12 IU; vitamin K_3_, 2.40 mg; vitamin B_1_, 1.80 mg; vitamin B_2_, 9.60 mg; vitamin B_6_, 3.60 mg; vitamin B_12_, 0.024 mg; nicotinic acid, 36 mg; pantothenic acid, 12 mg; folic acid, 1.20 mg; biotin, 0.240 mg; choline chloride, 1 000 mg; ferrous, 72 mg; copper, 9.60 mg; manganese, 120 mg; zinc, 48 mg; iodide, 0.360 mg; selenium, 0.240 mg.

2 Nutrient levels were calculated values.

### Growth performance

Ducks from each replicate were weighed on days 1 and 63, and the feed intake was recorded daily throughout the experiment to monitor consumption patterns and promptly identify any abnormal fluctuations that might indicate health issues or recording errors. From these data, the average daily feed intake (ADFI), average daily gain (ADG), and feed conversion ratio (FCR) were calculated.

### Carcass traits and sample collection

On day 63, two ducks that were close to the mean body weight of each replicate were selected and approximately 5 mL of blood was collected from the wing vein using sterile syringes. The blood samples were then centrifuged at 835 × *g* for 15 min and stored at −20°C. Thereafter, the birds were euthanized by exsanguination via the jugular vein. Following the procedure outlined in Performance Terminology and Measurements for Poultry (NY/T 823-2020), slaughter weight, semi-eviscerated weight, eviscerated weight, breast muscle weight, leg muscle weight, and abdominal fat weight were measured. Subsequently, as a percentage, slaughter rate, semi-eviscerated rate, eviscerated rate, breast muscle rate, leg muscle rate and abdominal fat rate were calculated. Finally, liver and breast muscle tissues were sampled by excising approximately 20 cm × 1.5 cm × 1.5 cm sections, which were then snap-frozen in liquid nitrogen and stored at −80°C for subsequent analysis of antioxidant status and 16S rRNA gene sequencing, respectively.

### Meat quality

Following slaughter, meat quality analysis was performed on breast and leg muscles from the right side of the carcass. All procedures followed the Determination of Meat Quality for Livestock and Poultry (NY/T 1333-2007) and the Determination of Meat Tenderness Shear Force Method (NY/T 1180-2006) guidelines. The shear force was determined using a digital tenderness meter (Jinan Saicheng Electronic Technology Co., Ltd., Jinan, China). The pH values (at 45 min and 24 h postmortem) were measured with a pH meter (HI8424, Beijing Hanna Instrument Technology Co., Ltd., Beijing, China). The meat color was measured at 45 min and 24 h postmortem using a colorimeter (OPTO-STAR, Beijing Bulader Technology Development Co., Ltd., Beijing, China), and expressed as L* (lightness), a* (redness), and b* (yellowness). Additionally, the crude fat content was determined in accordance with the Feed Analysis and Quality Test Technology described by Zhang (2004). Drip loss was evaluated using a standardized suspension technique. Briefly, samples (20 cm × 1.5 cm × 1.5 cm) from the leg and breast muscles were weighed, suspended in plastic bags for 24 h, and then reweighed. The drip loss was calculated based on the percentage mass difference as follows:

$$thedriploss\left(\%\right)=\frac{initialweight-finialweight}{initialweitght}\times 100\%$$

### Real-time PCR analysis of gene expression

Total RNA was extracted from liver and breast muscle tissues with SteadyPure RNA Extraction Kit (Hunan Aikerui Bioengineering Co., Ltd., Changsha, China). The concentration and purity of extracted RNA were assessed on NanoDrop 2000 (Thermo Fisher Scientific Corporation, Wilmington, NC, USA). Subsequently, total RNA was subjected to reverse transcription using Hifair® Ⅲ 1st Strand cDNA Synthesis SuperMix for qPCR (Yeasen Biotechnology Co., Ltd., Shanghai, China‌). The expression of relevant genes was detected by quantitative real-time PCR (qRT-PCR) under a fluorescence quantitative instrument (Bio-Rad Laboratories Co., Ltd., Shanghai, China). The procedure was as follows: initial denaturation at 95 °C for 5 min, followed by 40 cycles of denaturation at 95°C for 10 s, annealing at 55-56 °C for 10 s, and extension at 72 °C for 10 s. The primers were designed and synthesized by Shanghai Sangong Biological Engineering Co., Ltd., and the primer sequences are shown in Table 2. β-actin served as an internal control for normalization, and the relative expression of the target genes was determined using the 2^-ΔΔCt^ analysis method.

**Table 2:** Primer sequences of reference and target genes.

Gene	Primer Sequence
*ACTB*	F:5ʹ- CCAGCACGATGAAAATCAAGATCA −3ʹ
	R:5ʹ- TTGTCACAAGGGTGTGGGTG −3ʹ
*FASN*	F:5ʹ- GCCTGCCACAACTCTGAAGATAC −3ʹ
	R:5ʹ- CTCCTTTGCGAACACACCATCC −3ʹ
*ACC*	F:5ʹ- CCCCATCTCCACGAGGTTTT −3ʹ
	R:5ʹ- GTTAGGGGCAGTCACACCAA −3ʹ
*CPT-1*	F:5ʹ- GGGGAGATCCCTCCCATGAT −3ʹ
	R:5ʹ- CCGTAGTACAGCCACACCTT −3ʹ
*HMGCR*	F:5ʹ- CAAGAGCAAGTGCGTTAGCC −3ʹ
	R:5ʹ- AGTTGTCGCACACCTGACAT −3ʹ
*PRKAA1*	F:5ʹ- CTTCGGCAAAGTCAAGGTTGG −3ʹ
	R:5ʹ- AGGTTCTGAATCTCTCTGCGG −3ʹ
*SREBP-1C*	F:5ʹ- CATGGCAAGGTGAAGCAGGAG-3ʹ
	R:5ʹ- TTGAAGGAGAGGCAGAGGAAGAC −3ʹ
*ACTB*	F:5ʹ- CCAGCACGATGAAAATCAAGATCA −3ʹ
	R:5ʹ- TTGTCACAAGGGTGTGGGTG −3ʹ
*CAT*	F:5ʹ- TGTGCGTGACTGACAACCAAGG −3ʹ
	R:5ʹ- ACATGCGGCTCTCCTTCACAAC −3ʹ
*SOD*	F:5ʹ- AAAGGATGCAGAGAGGCACG −3ʹ
	R:5ʹ- GATGCAGTGTGGTCCAGTCA −3ʹ
*GPX*	F:5ʹ- ACTTCCTGCAGCTCAACGAG −3ʹ
	R:5ʹ- TTGGTGGCATTCTCCTGGTG −3ʹ
*KEAP1*	F:5ʹ- GCCTACACCGCCTCCATCTC −3ʹ
	R:5ʹ- AGCTGCTGCACCAGGAAGTC −3ʹ
*NRF2*	F:5ʹ- AATGGTTCCTGCTCAGATTGATAGTG −3ʹ
	R:5ʹ- GCATATTCTCCGCATCAGTAAGTGG −3ʹ
*NQO1*	F:5ʹ- CGTCGCCGAGCAGAAGAAGATC −3ʹ
	R:5ʹ- CTGGTGGTGAACGACAGCATGG −3ʹ
*HO1*	F:5ʹ- AAGAGCCAGGAGAACGGTCACC −3ʹ
	R:5ʹ- TGCCCACCAGGTCTGTCTGAC −3ʹ
*GLRX*	F:5ʹ- TTGGGACAACCTGCATTGGA −3ʹ
	R:5ʹ- TCGTGGTCATCTTTGTCCCTTC −3ʹ
*GCLC*	F:5ʹ- GGGCTGCTGTCGCAGG −3ʹ
	R:5ʹ- GCATATACTCCACCTCGTCGC −3ʹ
*TXN*	F:5ʹ- AGTTGACTTCTCGGCCACAT −3ʹ
	R:5ʹ- AGTGTGTAGCAACATCCTGGG −3ʹ

### Data analysis

Statistical analyses were performed using SPSS, version 27.0 (SPSS, Inc., Chicago, IL, USA). The experimental unit was the replicate pen, with each treatment group consisting of six replicates. Before analysis, data were tested for normality using the Shapiro–Wilk test and for homogeneity of variances using Levene's test. All data met the assumptions of normality and homogeneity of variance. The statistical significance of the results was analyzed by one-way analysis of variance. Where significant effects were found, all pairwise comparisons were conducted using Tukey’s multiple range tests for multiple comparisons. Results are reported as the mean ± standard deviation. Statistical significance was defined as *P* < 0.05.

## Results

### Growth performance

As shown in Table 3, dietary supplementation with HWE-JGLR had no effects on the ADFI, ADG, F/G and DR of Liancheng white ducks compared with the control group.

**Table 3:** Effects of HWE-JGLR on growth performance of Liancheng white ducks.

Items	Groups^1^					*P*-values		
	CON	HJ-Ⅰ	HJ-Ⅱ	HJ-Ⅲ	SEM^2^	ANOVA	Linear	Quadratic
ADFI (g)	61.1	64.6	63.0	61.9	1.11	0.205	0.617	0.088
ADG (g)	16.2	16.8	16.9	16.5	0.218	0.172	0.288	0.051
FCR	3.68	3.65	3.57	3.54	0.090	0.753	0.685	0.978

Abbreviations: HWE-JGLR, hot water extract of Juncao-substrate *Ganoderma lucidum* residue; ADFI, average daily feed intake; ADG, average daily gain; FCR, feed conversion ratio.

1 Control group, basal diet; HJ-Ⅰ group, basal diet added with 0.25% HWE-JGLR; HJ-Ⅱ group, basal diet added with 0.5% HWE-JGLR; HJ-Ⅲ group, basal diet with 1% HWE-JGLR.

2 Values are means with pooled SEM (*n* = 12, with 2 birds per replicate pen).

### Carcass traits

As shown in Table 4, the slaughter rate, semi-eviscerated rate, eviscerated rate, breast muscle rate, leg muscle rate and abdominal fat rate were not significantly affected by dietary HWE-JGLR supplementation.

**Table 4:** Effects of HWE-JGLR on carcass traits of 63-day-old Liancheng white ducks.

Items	Groups^1^					*P*-values		
	CON	HJ-Ⅰ	HJ-Ⅱ	HJ-Ⅲ	SEM^2^	ANOVA	Linear	Quadratic
Slaughter rate (%)	91.2	91.5	91.8	91.4	0.153	0.137	0.279	0.052
Semi-eviscerated rate (%)	83.8	82.4	81.5	81.6	0.748	0.217	0.065	0.336
Eviscerated rate (%)	71.6	71.2	70.3	69.7	0.865	0.544	0.162	0.970
Breast muscle rate (%)	7.89	8.58	7.12	7.66	0.393	0.143	0.270	0.867
Leg muscle rate (%)	11.9	11.8	11.0	11.2	0.308	0.177	0.057	0.717
Abdominal fat rate (%)	1.95	1.80	1.61	1.63	0.093	0.056	0.011	0.387

Abbreviations: HWE-JGLR, hot water extract of Juncao-substrate *Ganoderma lucidum* residue.

1 Control group, basal diet; HJ-Ⅰ group, basal diet added with 0.25% HWE-JGLR; HJ-Ⅱ group, basal diet added with 0.5% HWE-JGLR; HJ-Ⅲ group, basal diet with 1% HWE-JGLR.

2 Values are means with pooled SEM (*n* = 12, with 2 birds per replicate pen).

### Meat quality

As shown in Table 5, Table 6, dietary HWE-JGLR supplementation increased pH at 24 h in breast muscle (*P* < 0.05). Specifically, the 45 min postmortem pH of breast muscle was significantly higher in the HJ-Ⅱ and HJ-Ⅲ groups (6.38 and 6.22, respectively) than in the control group (5.96) (*P* < 0.05), indicating that HWE-JGLR mitigated the post-mortem pH decline in the breast muscle. In contrast, it had no significant effect on the shear force, drip loss, or meat color of the breast and leg muscles of the Liancheng white duck across all groups.

**Table 5:** Effects of HWE-JGLR on breast meat quality of 63-day-old Liancheng white ducks.

Items	Groups^1^					*P*-values		
	CON	HJ-Ⅰ	HJ-Ⅱ	HJ-Ⅲ	SEM^2^	ANOVA	Linear	Quadratic
Shear force (kgf)	3.27	3.26	3.06	3.00	0.565	0.799	0.353	0.895
Drip loss (%)	3.36	2.21	2.30	2.48	0.338	0.113	0.117	0.078
Crude fat (%)	2.15^a^	207^ab^	205^ab^	1.83^b^	0.075	0.043	0.009	0.371
pH_45 min_	5.96^b^	6.14^ab^	6.38^a^	6.22^a^	0.158	0.002	0.002	0.018
pH_24 h_	5.86^b^	6.11^a^	6.23^a^	6.18^a^	0.058	0.001	<0.001	0.018
Meat Color_45 min_								
L*	37.5	34.3	35.8	36.2	1.18	0.327	0.603	0.148
a*	11.8	11.9	12.9	11.7	0.883	0.731	0.852	0.451
b*	4.96	4.47	4.66	4.99	0.183	0.333	0.798	0.084
Meat Color_24 h_								
L*	40.2	37.6	40.0	39.5	1.05	0.383	0.926	0.389
a*	10.6	11.1	11.1	10.5	0.430	0.680	0.833	0.242
b*	5.93	5.06	5.36	5.55	0.460	0.609	0.694	0.263

Abbreviations: HWE-JGLR, hot water extract of Juncao-substrate *Ganoderma lucidum* residue; L*, lightness; a*, redness; b*, yellowness.

1 Control group, basal diet; HJ-Ⅰ group, basal diet added with 0.25% HWE-JGLR; HJ-Ⅱ group, basal diet added with 0.5% HWE-JGLR; HJ-Ⅲ group, basal diet with 1% HWE-JGLR.

2 Values are means with pooled SEM (*n* = 12, with 2 birds per replicate pen). Mean values within a row with different superscript letters denote statistically significant differences, *P* < 0.05.

**Table 6:** Effects of HWE-JGLR on leg meat quality of 63-day-old Liancheng white ducks.

Items	Groups^1^					*P*-values		
	CON	HJ-Ⅰ	HJ-Ⅱ	HJ-Ⅲ	SEM^2^	ANOVA	Linear	Quadratic
Shear force (kgf)	3.15	3.38	2.63	3.09	0.453	0.091	0.303	0.567
Drip loss (%)	2.30	2.15	2.66	2.12	0.295	0.591	0.991	0.540
Crude fat (%)	2.23	2.16	208	2.12	0.090	0.700	0.332	0.555
pH_45 min_	5.83	6.10	6.10	6.15	0.203	0.081	0.027	0.231
pH_24 h_	5.76	5.92	6.02	6.02	0.090	0.292	0.077	0.476
Meat Color_45 min_								
L*	39.3	39.0	37.4	38.8	0.930	0.576	0.519	0.398
a*	11.2	12.7	11.7	11.6	0.612	0.467	0.975	0.242
b*	6.78	6.87	5.93	5.90	0.613	0.582	0.230	0.923
Meat Color_24 h_								
L*	41.1	41.3	38.0	39.6	1.49	0.393	0.268	0.633
a*	11.1	13.1	11.7	13.0	0.595	0.070	0.113	0.578
b*	6.07	5.88	5.99	5.75	0.108	0.276	0.123	0.828

Abbreviations: HWE-JGLR, hot water extract of Juncao-substrate *Ganoderma lucidum* residue; L*, lightness; a*, redness; b*, yellowness.

1 Control group, basal diet; HJ-Ⅰ group, basal diet added with 0.25% HWE-JGLR; HJ-Ⅱ group, basal diet added with 0.5% HWE-JGLR; HJ-Ⅲ group, basal diet with 1% HWE-JGLR.

2 Values are means with pooled SEM (*n* = 12, with 2 birds per replicate pen).

### Lipid metabolism and AMPK pathway-related gene expression in breast muscle

**Fig. 1:**
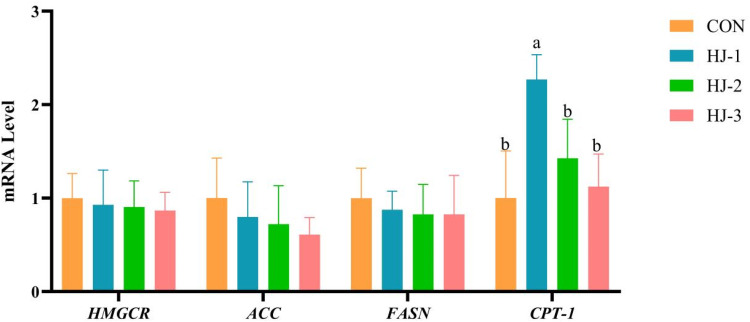
Effects of HWE-JGLR on lipid metabolism-related gene expression in the breast muscle of 63-day-old Liancheng white ducks. Abbreviations: HWE-JGLR, hot water extract of Juncao-substrate *Ganoderma lucidum* residue; HMGCR, 3‑hydroxy‑3-methylglutaryl-coenzyme A reductase; ACC, acetyl-CoA carboxylase; FASN, fatty acid synthase; CPT-1, carnitine palmitoyltransferase 1. Control group, basal diet; HJ-Ⅰ group, basal diet added with 0.25% HWE-JGLR; HJ-Ⅱ group, basal diet added with 0.5% HWE-JGLR; HJ-Ⅲ group, basal diet with 1% HWE-JGLR. Mean values within a row with different letters denote statistically significant differences, *P* < 0.05.

**Fig. 2:**
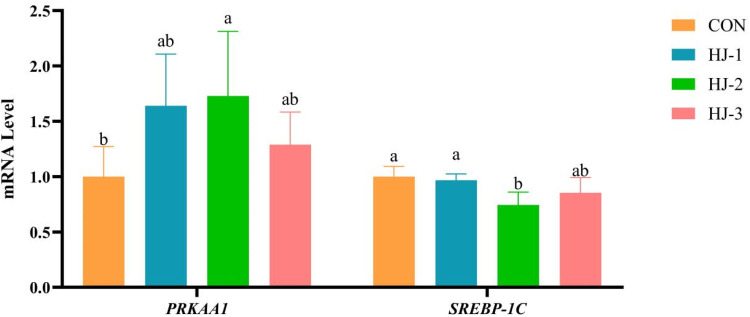
Effects of HWE-JGLR on AMPK pathway-related gene expression in the breast muscle of 63-day-old Liancheng white ducks. Abbreviations: HWE-JGLR, hot water extract of Juncao-substrate *Ganoderma lucidum* residue; PRKAA1, AMP-activated protein kinase α1 catalytic subunit; SREBP-1C, sterol regulatory element-binding protein 1c. Control group, basal diet; HJ-Ⅰ group, basal diet added with 0.25% HWE-JGLR; HJ-Ⅱ group, basal diet added with 0.5% HWE-JGLR; HJ-Ⅲ group, basal diet with 1% HWE-JGLR. Mean values within a row with different letters denote statistically significant differences, *P* < 0.05.

As shown in Fig. 1, Fig. 2, dietary HWE-JGLR supplementation had no significant effect on the expression of *HMGCR, ACC* and *FASN* genes in all groups. Besides, upregulation of *CPT-1* in the breast muscle was greater in the HJ-Ⅰ group than in the control (*P* < 0.05). A significant upregulation of *PRKAA1* expression was observed in the HJ-Ⅱ group, which was accompanied by a concurrent downregulation of *SREBP-1C* (*P* < 0.05).

### Antioxidant status in the serum, liver and breast muscle

As shown in Table 7, Table 8, Table 9, the concentrations of the serum T-SOD and GSH-Px in HJ-Ⅱ and HJ-Ⅲ groups were found to be elevated (*P* < 0.05). Furthermore, the content of serum MDA in the HJ-Ⅰ and HJ-Ⅲ groups was significantly lower than that of the control group (*P* < 0.05). Among the liver antioxidant status examined, only the content of GSH-Px in the HJ-Ⅱ group demonstrated a significant increase (*P* < 0.05). In comparison with the control group, the breast muscle CAT of the HJ-Ⅱ and HJ-Ⅲ Liancheng white ducks at 63 d of age resulted in a significant increase (*P* < 0.05), and the dietary supplementation of 0.25% HWE-JGLR significantly increased the breast muscle T-SOD compared with the control group (*P* < 0.05). However, Table 8 the content of CAT in serum and liver and the content of GSH-Px, MDA in breast muscle and γ-GCS, GSH, T-AOC among all the groups showed no significant effects.

**Table 7:** Effects of HWE-JGLR on serum antioxidant status of 63-day-old Liancheng white ducks.

Items	Groups^1^					*P*-values		
	CON	HJ-Ⅰ	HJ-Ⅱ	HJ-Ⅲ	SEM^2^	ANOVA	Linear	Quadratic
CAT (U/mL)	47.7	50.4	47.6	53.1	204	0.282	0.198	0.537
T-SOD (U/mL)	160^c^	164^c^	177^b^	184^a^	3.89	0.002	<0.001	0.729
GSH-Px (U/mL)	256^b^	254^b^	268^a^	272^a^	3.34	0.003	<0.001	0.398
GSH (μmol/mL)	18.8	21.7	18.5	19.3	1.39	0.423	0.788	0.491
MDA (nmol/mL)	4.22^a^	3.29^b^	3.90^a^	3.03^b^	0.188	<0.001	0.002	0.867
T-AOC (mmol/L)	0.623	0.674	0.661	0.651	0.020	0.438	0.493	0.184

Abbreviations: HWE-JGLR, hot water extract of Juncao-substrate *Ganoderma lucidum* residue; CAT, catalase; T-SOD, total superoxide dismutase; GSH-Px, glutathione peroxidase; GSH, glutathione; MDA, malondialdehyde; T-AOC, total antioxidant capacity.

1 Control group, basal diet; HJ-Ⅰ group, basal diet added with 0.25% HWE-JGLR; HJ-Ⅱ group, basal diet added with 0.5% HWE-JGLR; HJ-Ⅲ group, basal diet with 1% HWE-JGLR.

2 Values are means with pooled SEM (*n* = 12, with 2 birds per replicate pen). Mean values within a row with different superscript letters denote statistically significant differences, *P* < 0.05.

**Table 8:** Effects of HWE-JGLR on liver antioxidant status of 63-day-old Liancheng white ducks.

Items	Groups^1^					*P*-values		
	CON	HJ-Ⅰ	HJ-Ⅱ	HJ-Ⅲ	SEM^2^	ANOVA	Linear	Quadratic
CAT (U/mg prot)	23.9	27.8	23.9	26.0	1.25	0.119	0.693	0.479
T-SOD (U/mg prot)	203	214	182	205	13.1	0.622	0.726	0.722
GSH-Px (U/mg prot)	117^b^	119^b^	135^a^	120^b^	4.05	0.018	0.195	0.054
γ-GCS (U/mg prot)	5.47	5.73	5.97	5.65	0.158	0.201	0.273	0.097
GSH (μmol/mg prot)	49.7	52.6	58.2	59.5	2.27	0.072	0.010	0.868
MDA (nmol/mg prot)	0.467	0.351	0.355	0.393	0.038	0.311	0.341	0.102
T-AOC (mmol/g prot)	67.3	71.5	71.6	69.5	2.28	0.518	0.518	0.187

Abbreviations: HWE-JGLR, hot water extract of Juncao-substrate *Ganoderma lucidum* residue; CAT, catalase; T-SOD, total superoxide dismutase; GSH-Px, glutathione peroxidase; γ-GCS, γ-glutamylcysteine synthetase; GSH, glutathione; MDA, malondialdehyde; T-AOC, total antioxidant capacity.

1 Control group, basal diet; HJ-Ⅰ group, basal diet added with 0.25% HWE-JGLR; HJ-Ⅱ group, basal diet added with 0.5% HWE-JGLR; HJ-Ⅲ group, basal diet with 1% HWE-JGLR.

2 Values are means with pooled SEM (*n* = 12, with 2 birds per replicate pen). Mean values within a row with different superscript letters denote statistically significant differences, *P* < 0.05.

**Table 9:** Effects of HWE-JGLR on breast muscle antioxidant status of 63-day-old Liancheng white ducks.

Items	Groups^1^					*P*-values		
	CON	HJ-Ⅰ	HJ-Ⅱ	HJ-Ⅲ	SEM^2^	ANOVA	Linear	Quadratic
CAT (U/mg prot)	9.26^b^	9.87^ab^	11.24^a^	11.57^a^	0.488	0.015	0.002	0.785
T-SOD (U/mg prot)	104^b^	146^a^	105^b^	110^b^	2.69	<0.001	0.107	<0.001
GSH-Px (U/mg prot)	30.4	30.9	33.1	32.5	1.15	0.360	0.131	0.664
γ-GCS (U/mg prot)	3.96	4.46	4.71	4.61	0.280	0.303	0.110	0.310
GSH (μmol/mg prot)	37.5	45.0	40.5	43.4	2.76	0.284	0.307	0.428
MDA (nmol/mg prot)	1.65	1.36	1.46	1.41	0.085	0.138	0.139	0.186
T-AOC (mmol/g prot)	45.5	46.3	45.4	47.9	0.863	0.235	0.141	0.381

Abbreviations: HWE-JGLR, hot water extract of Juncao-substrate *Ganoderma lucidum* residue; CAT, catalase; T-SOD, total superoxide dismutase; GSH-Px, glutathione peroxidase; γ-GCS, γ-glutamylcysteine synthetase; GSH, glutathione; MDA, malondialdehyde; T-AOC, total antioxidant capacity.

1 Control group, basal diet; HJ-Ⅰ group, basal diet added with 0.25% HWE-JGLR; HJ-Ⅱ group, basal diet added with 0.5% HWE-JGLR; HJ-Ⅲ group, basal diet with 1% HWE-JGLR.

2 Values are means with pooled SEM (*n* = 12, with 2 birds per replicate pen). Mean values within a row with different superscript letters denote statistically significant differences, *P* < 0.05.

### Antioxidant-related gene expression in liver and breast muscle

**Fig. 3:**
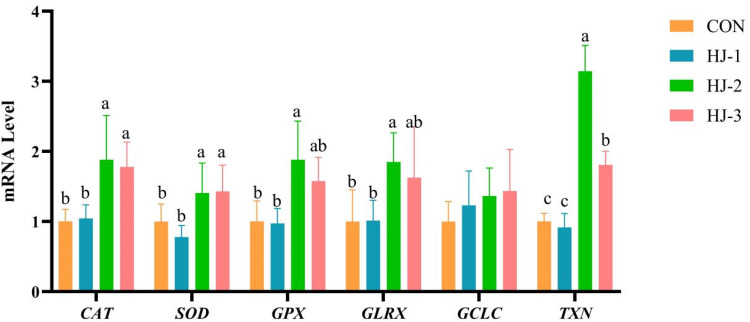
Effects of HWE-JGLR on antioxidant-related gene expression in the liver of 63-day-old Liancheng white ducks. Abbreviations: HWE-JGLR, hot water extract of Juncao-substrate *Ganoderma lucidum* residue; CAT, catalase; SOD, superoxide dismutase; GPX, glutathione peroxidase; GLRX, glutaredoxin; GCLC, glutamate-cysteine ligase catalytic subunit; TXN, thioredoxin. Control group, basal diet; HJ-Ⅰ group, basal diet added with 0.25% HWE-JGLR; HJ-Ⅱ group, basal diet added with 0.5% HWE-JGLR; HJ-Ⅲ group, basal diet with 1% HWE-JGLR. Mean values within a row with different letters denote statistically significant differences, *P* < 0.05.

**Fig. 4:**
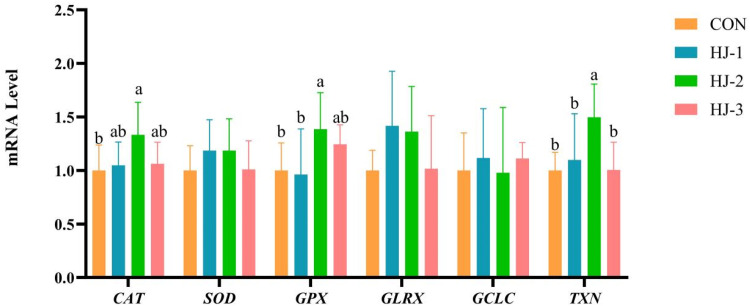
Effects of HWE-JGLR on antioxidant-related gene expression in the breast muscle of 63-day-old Liancheng white ducks. Abbreviations: HWE-JGLR, hot water extract of Juncao-substrate *Ganoderma lucidum* residue; CAT, catalase; SOD, superoxide dismutase; GPX, glutathione peroxidase; GLRX, glutaredoxin; GCLC, glutamate-cysteine ligase catalytic subunit; TXN, thioredoxin. Control group, basal diet; HJ-Ⅰ group, basal diet added with 0.25% HWE-JGLR; HJ-Ⅱ group, basal diet added with 0.5% HWE-JGLR; HJ-Ⅲ group, basal diet with 1% HWE-JGLR. Mean values within a row with different letters denote statistically significant differences, *P* < 0.05.

As shown in Fig. 3, Fig. 4, the HJ-Ⅱ group exhibited a significant upregulation in the expression of *CAT, SOD, GCLC, TXN, GPX* and *GLRX* genes in the liver of 63-day-old Liancheng white ducks when compared to the control group (*P* < 0.05). A similar upregulation was observed in the expression of *CAT, GPX* and *TXN* genes in breast muscle (*P* < 0.05). The mRNA expression levels of *CAT, SOD, GCLC* and *TXN* in HJ-Ⅲ were significantly increased (*P* < 0.05), while no significant differences were observed regarding the expression of antioxidant-related genes in breast muscle. In addition, the four groups exhibited comparable expression levels of *SOD, GLRX* and *GCLC* in breast muscle.

### Relative expression of genes related to the antioxidant signaling pathway Keap1-Nrf2/ARE

**Fig. 5:**
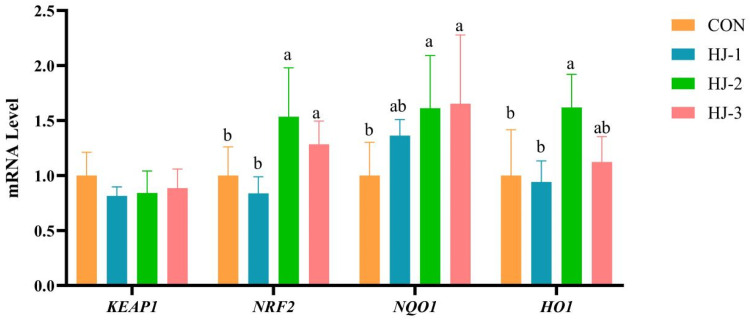
Effects of HWE-JGLR on KEAP1-NRF2/ARE pathway-related gene expression in the liver of 63-day-old Liancheng white ducks. Abbreviations: HWE-JGLR, hot water extract of Juncao-substrate *Ganoderma lucidum* residue; KEAP1, kelch-like ECH-associated protein 1; NRF2, nuclear factor erythroid 2-related factor 2; NQO1, NAD(P)H quinone **oxidoreductase 1**; HO1, heme oxygenase 1. Control group, basal diet; HJ-Ⅰ group, basal diet added with 0.25% HWE-JGLR; HJ-Ⅱ group, basal diet added with 0.5% HWE-JGLR; HJ-Ⅲ group, basal diet with 1% HWE-JGLR. Mean values within a row with different letters denote statistically significant differences, *P* < 0.05.

**Fig. 6:**
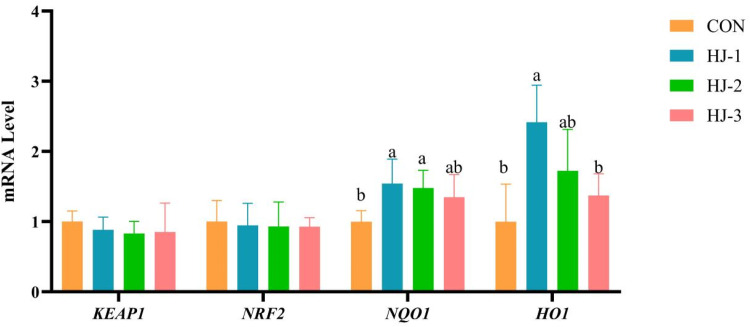
Effects of HWE-JGLR on KEAP1-NRF2/ARE pathway-related gene expression in the breast muscle of 63-day-old Liancheng white ducks. Abbreviations: HWE-JGLR, hot water extract of Juncao-substrate *Ganoderma lucidum* residue; KEAP1, kelch-like ECH-associated protein 1; NRF2, nuclear factor erythroid 2-related factor 2; NQO1, NAD(P)H quinone **oxidoreductase 1**; HO1, heme oxygenase 1. Control group, basal diet; HJ-Ⅰ group, basal diet added with 0.25% HWE-JGLR; HJ-Ⅱ group, basal diet added with 0.5% HWE-JGLR; HJ-Ⅲ group, basal diet with 1% HWE-JGLR. Mean values within a row with different letters denote statistically significant differences, *P* < 0.05.

It can be seen from Fig. 5, Fig. 6 that compared with the control group, the HJ-Ⅱ and HJ-Ⅲ groups significantly up-regulated the expression of the liver *NRF2* and *NQO1* (*P* < 0.05), with the HJ-Ⅱ also exhibiting a significant increase in *HO1* expression (*P* < 0.05). The breast muscle *NQO1* expression of HJ-Ⅰ and HJ-Ⅱ groups was significantly higher than that of the control group (*P* < 0.05). Additionally, the expression of breast muscle *HO1* was significantly increased in the HJ-Ⅰ group (*P* < 0.05). Conversely, the expression of *NRF2* in breast muscle was found to be unaffected. There were no significant differences in either tissue.

## Discussion

The growth performance of livestock and poultry directly impacts breeding efficiency and economic benefits (Wang et al., 2024). Superior growth performance contributes to enhanced product quality, meeting market demands for high-quality products (Niu et al., 2022). Recent studies confirm that GLP, the primary active component of HWE-JGLR, improves both intestinal health and growth performance (Fang et al., 2025). However, this growth-promoting effect is not universally observed. Martínez et al. (2022) reported no significant effect on growth performance from adding 2.5 g/kg of *Ganoderma lucidum* to the diet of 22 to 50-day-old broilers under their experimental conditions. Likewise, Chen and Yu (2020) also observed no significant improvement in growth performance following the administration of 1 g/L *Ganoderma lucidum* extract in broiler chickens aged 21 to 35 days. Consistent with these reports, our study also found that dietary HWE-JGLR supplementation had no significant effect on the ADFI, ADG, or F/G of 63-day-old Liancheng white ducks. The disparity between the potential activity of GLP and the observed lack of growth enhancement in our and others' studies may be attributed to its pharmacokinetic profile. One possible explanation is that GLP may resist digestion in the upper gastrointestinal tract and instead undergoes extensive microbial degradation in the large intestine, thereby limiting its efficacy (Ding et al., 2017). Furthermore, species-specific physiological differences might play a certain role. It could be hypothesized that the potentially higher metabolic rate of Liancheng white ducks may lead to accelerated clearance of GLP (Miao et al., 2014). This would prevent GLP from reaching or sustaining the effective concentrations required within the necessary therapeutic window to exert a measurable effect on growth performance.

Slaughter performance serves as a critical indicator in livestock production, reflecting the yield of premium meat cuts and nutrient partitioning (Ding et al., 2021). A higher intramuscular fat content imparts superior flavor, tenderness, and juiciness to duck meat (Yang et al., 2022), whereas abdominal fat accumulation is often viewed as undesirable, thereby diminishing consumer purchasing desire (Zhang et al., 2017). Our results indicate that HWE-JGLR supplementation did not significantly affect the carcass traits in 63-day-old Liancheng white ducks. This absence of effect aligns with the report by Zheng et al. (Zheng et al., 2025), who observed no substantial changes in the carcass traits of chickens supplemented with Yupingfeng polysaccharide. Interestingly, our results revealed a decreasing trend in abdominal fat percentage. We hypothesize that this trend is linked to bioactive substances in HWE-JGLR, particularly GLP, which is well-documented to exert hypolipidemic (Wu, 2018) and hypoglycemic (Xiao et al., 2012) effects. These effects could promote a more favorable nutrient repartitioning, potentially by enhancing lipid catabolism or inhibiting lipogenesis in meat ducks.

Amidst the continuing expansion of large-scale livestock and poultry production, enhancing meat quality has emerged as a central concern for the industry (Mo et al., 2023), driven by its direct impact on consumer choice and market demand (Kirkpinar et al., 2014; Lee et al., 2022; Mir et al., 2017). Following slaughter, the cessation of circulating nutrient supply initiates glycolysis in muscle tissue, leading to lactic acid production and a concomitant decrease in muscle pH (Kiyimba et al., 2024), a key determinant of meat tenderness, drop loss and texture. This pH-stabilizing effect of dietary supplements is evidenced across studies. Dietary supplementation with 800 mg/kg of Ganoderma lucidum extracellular polysaccharides was shown by Liu et al. (2025) to significantly elevate post-slaughter pH at 45 min and 24 h, effectively countering the typical pH decline. Consistently, our results demonstrate that dietary supplementation with 0.5% and 1% HWE-JGLR effectively mitigated the breast muscle pH decline in Liancheng white ducks over the 63-day trial. We attribute this pH amelioration primarily to GLP's capacity to regulate glycolysis. By modulating the activity of key glycolytic enzymes, GLP has been shown to decelerate the rate of glycolysis in muscle tissue (Luo et al., 2022), thus effectively mitigating the subsequent pH decline. Furthermore, the lipid-modulating role of GLP is supported by our observation that 1% HWE-JGLR supplementation significantly reduced the crude fat content of breast muscle, corroborating its documented hypolipidemic effects and suggesting a broader role in nutrient partitioning.

As a key sensor of cellular energy status, AMPK orchestrates metabolic pathways to ensure energy homeostasis (Steinberg and Hardie, 2023). AMPK is activated upon depletion of cellular energy, a signal that is triggered by the phosphorylation of specific sites on its α subunit (Garcia and Shaw, 2017). Furthermore, AMPK is subject to phosphoactivation by upstream kinases, including *LKB1, CaMKKβ*, and *TAK1* (Neumann, 2018). Once activated, AMPK acts as a master regulator, orchestrating energy metabolic homeostasis. To drive energy production, AMPK enhances fatty acid oxidation through the phosphorylation-mediated activation of *CPT-1* and boosts glycolysis by activating *PFK-1* (Li et al., 2020). Conversely, AMPK shuts down energy-intensive processes such as lipid synthesis by phosphorylating and inhibiting *ACC*. A body of evidence demonstrates the efficacy of polysaccharides in ameliorating lipid metabolism disorders (Huang et al., 2022, 2023; Wu et al., 2024). Li et al. (2023b) reported that Ganoderma lucidum polysaccharides induce AMPK phosphorylation, which reduces both protein degradation and lipid accumulation. Furthermore, Lee et al. (2020) discovered that the anti-obesity activity of *Ganoderma lucidum* extract is mediated by the transcriptional downregulation of *FASN* and *SREBP-1C*. Consistent with this established mechanism, our study provides in vivo evidence that dietary HWE-JGLR supplementation elicited a differential regulation of key metabolic genes in the breast muscle of Liancheng white ducks. Specifically, we observed the upregulation of *CPT-1* and *PRKAA1* alongside downregulation of *HMGCR, FASN*, and *SREBP-1C*. These observations suggest that HWE-JGLR acts by activating the key AMPK subunit *PRKAA1*, thereby initiating this signaling pathway. Collectively, HWE-JGLR lowers cholesterol concentrations primarily through the inhibition of *HMGCR*. Moreover, it coordinately promotes lipid catabolism by suppressing *ACC* to reduce fatty acid synthesis, while concurrently activating *CPT-1* to promote fatty acid oxidation. The discrepancy between the observed transcriptional responses and the lack of significant effects on growth performance and carcass traits may be explained by factors such as limited intestinal absorption or rapid metabolic clearance of the bioactive components in HWE-JGLR, as discussed previously. Nonetheless, these molecular findings provide important mechanistic evidence that HWE-JGLR modulates lipid metabolism pathways in vivo. Furthermore, the elevation in post-slaughter pH suggests that these metabolic adjustments had measurable physiological consequences and may serve as an early indicator of potential benefits that could emerge with prolonged supplementation or optimized dosage regimens.

Reactive oxygen species (ROS) are oxygen-containing, chemically reactive molecules that originate from metabolic processes, cellular respiration, and other redox reactions as natural byproducts (Shafiq et al., 2021). When ROS concentrations overwhelm the body's antioxidant defenses, oxidative stress occurs. This imbalance leads to the oxidation of biomolecules such as proteins, lipids, and DNA, ultimately causing cellular damage and disease (Li et al., 2023a; Maldonado et al., 2023). Evidence suggests that GLP enhances antioxidant defense by directly scavenging free radicals (Tan et al., 2018; Wang et al., 2013) and stimulating the activity of antioxidant enzymes (Meng et al., 2011), thereby bolstering the body's capacity to neutralize ROS. When maintained at low homeostatic concentrations, intracellular ROS do not cause oxidative damage but instead serve as crucial signaling molecules in redox-regulated pathways (Santos et al., 2016). During this process, ROS can act directly on cysteine residues in *GLRX* and *TXN*, oxidizing their sulfhydryl (-SH) groups to promote the formation of disulfide bonds or other oxidation products, thereby regulating protein activity (Oh and Lee, 2016). In addition, low-level ROS can facilitate the release of active *GLRX* and *TXN* by promoting the dissociation of certain complexes (Sevilla et al., 2023). Building upon this foundation, our study provides novel insights at the gene expression level. We found that 0.25% HWE-JGLR, when added to the diet, significantly enhanced *GLRX* expression in the breast muscle of Liancheng white ducks. This finding suggests a targeted, mechanism-based enhancement of the cellular redox buffer system. *GLRX*, which uses GSH as a cofactor, works in concert with glutathione reductase (GR). GR recycles GSSG back to GSH, ensuring a continuous cellular reducing capacity (Matsui et al., 2017). In contrast, *TXN* can directly react with H₂O₂ (Kim et al., 2020), accepting its electrons and being oxidized into a disulfide form. It is then regenerated to its reduced state by TR and NADPH (Yang et al., 2017), forming a reversible cycle coupled to ROS scavenging. In light of the research findings outlined above, we hypothesized that HWE-JGLR augments cellular resilience to oxidative stress by supporting antioxidant capacity and improving electron transport efficiency, thus maintaining redox stability.

*NRF2* is a key transcription factor that orchestrates the cellular antioxidant response via the KEAP1-NRF2/ARE signaling pathway, governing the expression of a wide array of cytoprotective genes (Culletta et al., 2024). In the absence of stress, *NRF2* complexes with *KEAP1* in the cytoplasm, remaining in an inactive state (Ding, 2021). However, under oxidative stress, this equilibrium is disrupted, leading to *NRF2* activation. Liberated *NRF2* then translocates to the nucleus and upregulates a suite of antioxidant enzymes, including SOD, HO1, NQO1, GSH-Px and CAT (Tossetta and Marzioni, 2023). Together, these enzymes act synergistically to maintain cellular redox homeostasis. Polysaccharides are known to modulate the *NRF2*-mediated antioxidant pathway (Luo et al., 2023). By upregulating key genes, including *NRF2, HO1, GCL*C and *NQO1* in the *NRF2* signaling pathway, GLP alleviates oxidative stress, consequently suppressing hepatocyte necrosis and inflammatory cell infiltration (Ni et al., 2024; Zhang et al., 2024, 2021). In this study, HWE-JGLR conferred protection against oxidative stress via the *NRF2* pathway, sustaining the cellular redox balance through coordinated regulation of its downstream genes and proteins.

## Conclusion

In conclusion, dietary supplementation with HWE-JGLR in Liancheng white ducks mitigated the decline in pH and reduced the crude fat content in the breast muscle of Liancheng white ducks. Furthermore, the inclusion of HWE-JGLR in the diet could enhance the expression of *CPT-1* and *PRKA1*, while downregulating *SREBP-1C* in the breast muscle. Additionally, dietary supplementation with HWE-JGLR has been shown to enhance antioxidant enzyme activities and upregulate the expression of antioxidant-related genes, potentially through the activation of the *NRF2* signaling pathway. Considering the overall improvements observed, dietary supplementation with 0.5% HWE-JGLR is recommended for Liancheng white ducks. This study provides theoretical and practical evidence for the application of HWE-JGLR as a functional feed additive to improve meat quality and promote sustainable production practices in Liancheng white ducks.

## CRediT authorship contribution statement

**Zai-Xing Cai:** Writing – review & editing, Writing – original draft, Software, Investigation, Formal analysis. **Hai-Xuan Lv:** Software, Investigation. **Yun Yang:** Validation, Formal analysis, Data curation. **Xiao-Ming Gu:** Validation, Formal analysis. **Xiao-Ping Liu:** Investigation. **Ling Jin:** Supervision, Resources, Project administration. **Yu-Yun Gao:** Writing – review & editing, Methodology, Conceptualization.

## Disclosures

We declare that we have no financial and personal relationships with other people or organizations that can inappropriately influence our work, there is no professional or other personal interest of any nature or kind in any product, service or company that could be construed as influencing the position presented, or the review of,the manuscript entitled.

## References

[bib0001] Averós X., Estevez I. (2018). Meta-analysis of the effects of intensive rearing environments on the performance and welfare of broiler chickens. Poult. Sci..

[bib0002] Blundell R., Camilleri E., Baral B., Karpiński T.M., Neza E., Atrooz O.M. (2023). The phytochemistry of ganoderma species and their medicinal potentials. Am. J. Chin. Med..

[bib0003] Castanon J.I. (2007). History of the use of antibiotic as growth promoters in European poultry feeds. Poult. Sci..

[bib0004] Chen H.W., Yu Y.H. (2020). Effect of Ganoderma lucidum extract on growth performance, fecal microbiota, and bursal transcriptome of broilers. Anim. Feed Sci. Technol..

[bib0005] Cör D., Knez Ž., Knez Hrnčič M. (2018). Antitumour, antimicrobial, antioxidant and antiacetylcholinesterase effect of Ganoderma Lucidum terpenoids and polysaccharides: a review. Molecules.

[bib0006] Culletta G., Buttari B., Arese M., Brogi S., Almerico A.M., Saso L., Tutone M. (2024). Natural products as non-covalent and covalent modulators of the KEAP1/NRF2 pathway exerting antioxidant effects. Eur. J. Med. Chem..

[bib0007] Ding, Q., Nie, S., Hu, J., Zong, X., Li, Q., and Xie, M. 2017. In vitro and in vivo gastrointestinal digestion and fermentation of the polysaccharide from Ganoderma atrum. Food Hydrocoll. 63, 646-655 10.1016/j.foodhyd.2016.10.018.

[bib0008] Ding T. (2021).

[bib0009] Ding Y., Jiang X., Yao X., Zhang H., Song Z., He X., Cao R. (2021). Effects of feeding fermented mulberry leaf powder on growth performance, slaughter performance, and meat quality in chicken broilers. Animals.

[bib0010] Fang H., Yang S., Yang T. (2025). Ganoderma lucidum polysaccharides: a comprehensive overview of pharmacological effects and future perspectives. Food Biosci..

[bib0011] Gao J., Yang Z., Zhao C., Tang X., Jiang Q., Yin Y. (2023). A comprehensive review on natural phenolic compounds as alternatives to in-feed antibiotics. Sci. China Life Sci..

[bib0012] Gao Y.Y., Liu X.P., Zhou Y.H., He J.Y., Di B., Zheng X.Y., Guo P.T., Zhang J., Wang C.K., Jin L. (2024). The addition of hot water extract of juncao-substrate ganoderma lucidum residue to diets enhances growth performance, immune function, and intestinal health in broilers. Animals.

[bib0013] Garcia D., Shaw R.J. (2017). AMPK: mechanisms of cellular energy sensing and restoration of metabolic balance. Mol. Cell.

[bib0014] Gornowicz E., Dobek A., Moliński K., Szwaczkowski T. (2023). The quality of duck meat from the perspective of physical measurements and expert judgment. Ann. Anim. Sci..

[bib0015] Hermier D. (1997). Lipoprotein metabolism and fattening in poultry. J. Nutr..

[bib0016] Huang R., Wu E., Deng X. (2022). Potential of Lycium barbarum polysaccharide for the control of glucose and lipid metabolism disorders: a review. Int. J. Food Prop..

[bib0017] Huang Z., Ye Y., Long Z., Qin H., Liu L., Xu A., Li Z. (2023). Lycium barbarum polysaccharides improve lipid metabolism disorders of spotted sea bass lateolabrax maculatus induced by high lipid diet. Int. J. Biol. Macromol..

[bib0018] Kim M.J., Han C., White K., Park H.J., Ding D., Boyd K., Rothenberger C., Bose U., Carmichael P., Linser P.J., Tanokura M., Salvi R., Someya S. (2020). Txn2 haplodeficiency does not affect cochlear antioxidant defenses or accelerate the progression of cochlear cell loss or hearing loss across the lifespan. Exp. Gerontol..

[bib0019] Kirkpinar F., Ünlü H.B., Serdaroğlu M., Turp G.Y. (2014). Effects of dietary oregano and garlic essential oils on carcass characteristics, meat composition, colour, pH and sensory quality of broiler meat. Br. Poult. Sci..

[bib0020] Kiyimba F., Hartson S.D., Mafi G.G., Ramanathan R. (2024). Glycogen supplementation in vitro promotes pH decline in dark-cutting beef by reverting muscle's metabolome toward a normal postmortem muscle State. J. Agric. Food. Chem..

[bib0021] Kong M., Yao Y., Zhang H. (2019). Antitumor activity of enzymatically hydrolyzed Ganoderma lucidum polysaccharide on U14 cervical carcinoma-bearing mice. Int. J. Immunopathol. Pharmacol..

[bib0022] Lee H.A., Cho J.H., Afinanisa Q., An G.H., Han J.G., Kang H.J., Choi S.H., Seong H.A. (2020). Ganoderma lucidum extract reduces insulin resistance by enhancing AMPK activation in high-fat diet-induced obese mice. Nutrients.

[bib0023] Lee W.D., Kothari D., Moon S.G., Kim J., Kim K.I., Ga G.W., Kim Y.G., Kim S.K. (2022). Evaluation of non-fermented and fermented Chinese chive juice as an alternative to antibiotic growth promoters of broilers. Animals.

[bib0024] Li H., Yang N., Chen K., Chen G., Tang Q., Tu Y., Yu Y., Ma Y. (2006). Study on molecular genetic diversity of native duck breeds in China. World's Poult. Sci. J..

[bib0025] Li J., Pan L., Pan W., Li N., Tang B. (2023). Recent progress of oxidative stress associated biomarker detection. Chem. Commun..

[bib0026] Li J., Zhang Y., Yu F., Pan Y., Zhang Z., He Y., Yang H., Zhou P. (2023). Proteoglycan extracted from Ganoderma lucidum ameliorated diabetes-induced muscle atrophy via the AMPK/SIRT1 pathway In Vivo and In Vitro. ACS Omega.

[bib0027] Li X.N., Xu N., Zhao J.G., Liu Y.C., Zhao W.G., Hu Z.L. (2020). Recent anti-aging studies with caloric restriction and its mimetics. Prog. Physiol. Sci..

[bib0028] Li Y., Zhang C.C., Chen J. (2022). Application of fermented feed in livestock and poultry breeding under the background of prohibition and resistance. Mod. Anim. Husb. Sci. Technol..

[bib0029] Liu G., Zhang J., Kan Q., Song M., Hou T., An S., Lin H., Chen H., Hu L., Xiao J., Chen Y., Cao Y. (2022). Extraction, structural characterization, and immunomodulatory activity of a high molecular weight polysaccharide from Ganoderma lucidum. Front. Nutr..

[bib0030] Liu P., Zhu R., Gu Y., Xu Z., Zou H., Gu J., Yuan Y., Liu Z., Bian J. (2025). Effects of Ganoderma spent substrate containing with polysaccharides and triterpenoids on the growth performance, antioxidant capacity and immunity level of chickens. Int. J. Biol. Macromol..

[bib0031] Liu Y., Zhao C., Lin D., Lan H., Lin Z. (2015). Effects of ganoderma lucidum spent mushroom substrate extract on milk and serum immunoglobulin levels and serum antioxidant capacity of dairy cows. Trop. J. Pharm. Res..

[bib0032] Liu Y., Zhao C., Lin D., Lin H., Lin Z. (2015). Effect of water extract from spent mushroom substrate after Ganoderma balabacense cultivation by using JUNCAO technique on production performance and hematology parameters of dairy cows. Anim. Sci. J..

[bib0033] Liu Y.L., Li W.D., Lin D.M., Lin X.S., Lin Z.X. (2015). Serum antioxidant activity of hot water extract of spent mushroom substrate from Ganoderma lucidum cultivated by Juncao evaluated using an immune-deficient mouse model. J. Fujian Agric. For. Univ. Nat. Sci. Ed..

[bib0034] Liu Y.L., Liu P.H., Li J., Wang T., Qi B., Lin D.M., Lin Z.X. (2022). Growth and chemical composition of ganoderma lucidum as affected by cultivation substrates. Fujian J. Agric. Sci..

[bib0035] Luo J.H., Li J., Shen Z.C., Lin X.F., Chen A.Q., Wang Y.F., Gong E.S., Liu D., Zou Q., Wang X.Y. (2023). Advances in health-promoting effects of natural polysaccharides: regulation on Nrf2 antioxidant pathway. Front. Nutr..

[bib0036] Luo M., Liao B., Ma D., Wang J., Wang J., Liu J., Lei X., Cai Y., Tang L., Zhao L., Long S., Yang F., Lei X. (2022). Dendrobium nobile-derived polysaccharides ameliorate spermatogenic disorders in mice with streptozotocin-induced diabetes through regulation of the glycolytic pathway. Int. J. Biol. Macromol..

[bib0037] Maldonado E., Morales-Pison S., Urbina F., Solari A. (2023). Aging hallmarks and the role of oxidative stress. Antioxidants.

[bib0038] Martín C., Zervakis G.I., Xiong S., Koutrotsios G., Strætkvern K.O. (2023). Spent substrate from mushroom cultivation: exploitation potential toward various applications and value-added products. Bioengineered.

[bib0039] Martínez Y., Paredes J., Avellaneda M.C., Botello A., Valdivié M. (2022). Diets with Ganoderma lucidum mushroom powder and zinc-bacitracin on growth performance, carcass traits, lymphoid organ weights and intestinal characteristics in broilers. Braz. J. Poult. Sci..

[bib0040] Matsui R., Watanabe Y., Murdoch C.E. (2017). Redox regulation of ischemic limb neovascularization - what we have learned from animal studies. Redox Biol.

[bib0041] Meng G., Zhu H., Yang S., Wu F., Zheng H., Chen E., Xu J. (2011). Attenuating effects of Ganoderma lucidum polysaccharides on myocardial collagen cross-linking relates to advanced glycation end product and antioxidant enzymes in high-fat-diet and streptozotocin-induced diabetic rats. Carbohydr. Polym..

[bib0042] Miao Z.W., Zhu Z.M., Xin Q.W., Zheng N.Z., Zhuang X.D., Li D.S., Chen H. (2014). Slaughter performance and meat quality of new meat lines of Liancheng White duck. Fujian J. Agric. Sci..

[bib0043] Mir N.A., Rafiq A., Kumar F., Singh V., Shukla V. (2017). Determinants of broiler chicken meat quality and factors affecting them: a review. J. Food. Sci. Technol..

[bib0044] Mo M., Zhang Z., Wang X., Shen W., Zhang L., Lin S. (2023). Molecular mechanisms underlying the impact of muscle fiber types on meat quality in livestock and poultry. Front. Vet. Sci..

[bib0045] Nataraj A., Govindan S., Rajendran A., Ramani P., Subbaiah K.A., Munekata P.E.S., Pateiro M., Lorenzo J.M. (2023). Effects of carboxymethyl modification on the acidic polysaccharides from Calocybe indica: physicochemical properties. Antioxid. Antitumor Anticoagulant Act., Antioxid..

[bib0046] Neumann D. (2018). Is TAK1 a direct upstream kinase of AMPK?. Int. J. Mol. Sci..

[bib0047] Ni D.R., Li H.Y., Li Z.P., Liu J.W. (2024). In vitro evaluation of the antitumor and antioxidant effects of purified and characterized polysaccharides from Ganoderma applanatum. Ann. Med..

[bib0048] Niu X., Ding Y., Chen S., Gooneratne R., Ju X. (2022). Effect of immune stress on growth performance and immune functions of livestock: mechanisms and prevention. Animals.

[bib0049] Oh B., Lee C.H. (2016). Development of thiolated-graphene quantum dots for regulation of ROS in macrophages. Pharm Res..

[bib0050] Rousseau G. (2021). Microbiota, a new playground for the omega-3 polyunsaturated fatty acids in cardiovascular diseases. Mar. Drugs.

[bib0051] Santos C.X., Raza S., Shah A.M. (2016). Redox signaling in the cardiomyocyte: from physiology to failure. Int. J. Biochem. Cell. Biol..

[bib0052] Sevilla F., Martí M.C., De Brasi-Velasco S., Jiménez A. (2023). Redox regulation, thioredoxins, and glutaredoxins in retrograde signalling and gene transcription. J. Exp. Bot..

[bib0053] Seweryn E., Ziała A., Gamian A. (2021). Health-promoting of polysaccharides extracted from Ganoderma lucidum. Nutrients.

[bib0054] Shafiq M., Chen Y., Hashim R., He C., Mo X., Zhou X. (2021). Reactive oxygen species-based biomaterials for regenerative medicine and tissue engineering applications. Front. Bioeng. Biotechnol..

[bib0055] Steinberg G.R., Hardie D.G. (2023). New insights into activation and function of the AMPK. Nat. Rev. Mol. Cell Biol..

[bib0056] Tan X., Sun J., Xu Z., Li H., Hu J., Ning H., Qin Z., Pei H., Sun T., Zhang X. (2018). Effect of heat stress on production and in-vitro antioxidant activity of polysaccharides in Ganoderma lucidum. Bioprocess Biosyst. Eng..

[bib0057] Tossetta G., Marzioni D. (2023). Targeting the NRF2/KEAP1 pathway in cervical and endometrial cancers. Eur. J. Pharmacol..

[bib0058] Wang J., Wang Y., Liu X., Yuan Y., Yue T. (2013). Free radical scavenging and immunomodulatory activities of Ganoderma lucidum polysaccharides derivatives. Carbohydr. Polym..

[bib0059] Wang Z., Li Q., Yu Q., Qian W., Gao R., Wang R., Wu T., Li X. (2024). A review of visual estimation research on live pig weight. Sensors (Basel).

[bib0060] Wu L., Li Y., Chen S., Yang Y., Tang B., Weng M., Shen H., Chen J., Lai P. (2024). Widely targeted lipidomics and microbiomics perspectives reveal the mechanism of auricularia auricula polysaccharide's effect of regulating glucolipid metabolism in high-fat-diet mice. Foods.

[bib0061] Wu S. (2018). Hypolipidaemic and anti-lipidperoxidant activities of Ganoderma lucidum polysaccharide. Int. J. Biol. Macromol..

[bib0062] Xiao C., Wu Q.P., Cai W., Tan J.B., Yang X.B., Zhang J.M. (2012). Hypoglycemic effects of Ganoderma lucidum polysaccharides in type 2 diabetic mice. Arch. Pharm. Res..

[bib0063] Yang C., Wang Z., Song Q., Dong B., Bi Y., Bai H., Jiang Y., Chang G., Chen G. (2022). Transcriptome sequencing to identify important genes and lncRNAs regulating abdominal fat deposition in ducks. Animals.

[bib0064] Yang J., Hamid S., Cai J., Liu Q., Xu S., Zhang Z. (2017). Selenium deficiency-induced thioredoxin suppression and thioredoxin knock down disbalanced insulin responsiveness in chicken cardiomyocytes through PI3K/Akt pathway inhibition. Cell Signal.

[bib0065] Zhang N., Han Z., Zhang R., Liu L., Gao Y., Li J., Yan M. (2024). Ganoderma lucidum polysaccharides ameliorate acetaminophen-induced acute liver injury by inhibiting oxidative stress and apoptosis along the Nrf2 pathway. Nutrients.

[bib0066] Zhang T., Zhang X., Han K., Zhang G., Wang J., Xie K., Xue Q. (2017). Genome-wide analysis of lncRNA and mRNA expression during differentiation of abdominal preadipocytes in the chicken. G3 Bethesda.

[bib0067] Zhang Y., Feng Y., Wang W., Jia L., Zhang J. (2021). Characterization and hepatoprotections of ganoderma lucidum polysaccharides against multiple organ dysfunction syndrome in mice. Oxid. Med. Cell Longev..

[bib0068] Zheng W., Chen S., Guan Y., Wu B. (2025). Effects of Yupingfeng polysaccharide in diet on slaughtering performance and meat flavor of Qingyuan partridge chicken. Food Chem..

